# BRAF class II-III mutations in NSCLC: a single center experience

**DOI:** 10.3389/fonc.2026.1717614

**Published:** 2026-03-17

**Authors:** Sara Torresan, Martina Bortolot, Elisa Bertoli, Alessandro Del Conte, Brigida Stanzione, Elisa de Carlo, Monica Schiappacassi, Michele Spina, Gustavo Baldassarre, Alessandra Bearz

**Affiliations:** 1 Medical Oncology Department, CRO Aviano, National Cancer Institute, Istituto di Ricovero e Cura a Carattere Scientifico (IRCCS), Aviano, Italy; 2 Department of Medicine, University of Udine, Udine, Italy; 3 Molecular Oncology Unit, CRO Aviano, National Cancer Institute, Istituto di Ricovero e Cura a Carattere Scientifico (IRCCS), Aviano, Italy

**Keywords:** BRAF, class II mutations, non-small cell lung cancer, target therapy, TKI

## Abstract

**Background:**

Currently, there is no consensus on the optimal treatment sequence for NSCLC patients with class II-III *BRAF* mutations, where standard of care implies immunochemotherapy and scarce data on targeted therapy is available.

**Materials and methods:**

This is an observational, single centre case series of 9 patients collected in a Cancer Institute in Italy diagnosed with NSCLC with a *BRAF* class II or III mutation between 2020 and April 2025. All clinico-pathological data were anonymously collected with patient consent if they were alive at the time of data collection (2025). All patients were discussed by the Institutional Molecular Tumor Board. When feasible, off-label targeted therapy with dabrafenib and trametinib was administered. Data on type of BRAF mutation, variant allele frequency, co mutations, and clinical outcomes are reported. Progression-free survival was measured in patients treated with dabrafenib and trametinib; overall survival for all patients. Toxicities related to targeted therapy were graded using CTCAE v5.0.

**Results:**

The most common mutation was BRAF G469 (class IIb), observed in 4 of 9 patients. Median age was 76 years. Only three patients received dabrafenib and trametinib for longer than one month, achieving stable disease as the best response. Among these patients, median progression-free survival was 7.5 months and median overall survival was 18.7 months. Across the entire cohort, median overall survival was 16.6 months. Independently of the prior treatment received, only one patient experienced grade 3 treatment-related toxicity.

**Conclusions:**

This case series highlights the clinical heterogeneity and poor prognosis of NSCLC patients with *BRAF* class II-III mutations. While dabrafenib and trametinib were well tolerated, treatment feasibility was restricted. These findings emphasize the urgent need for more robust research to identify effective therapeutic strategies for this molecular subgroup.

## Introduction

**Figure 1:**
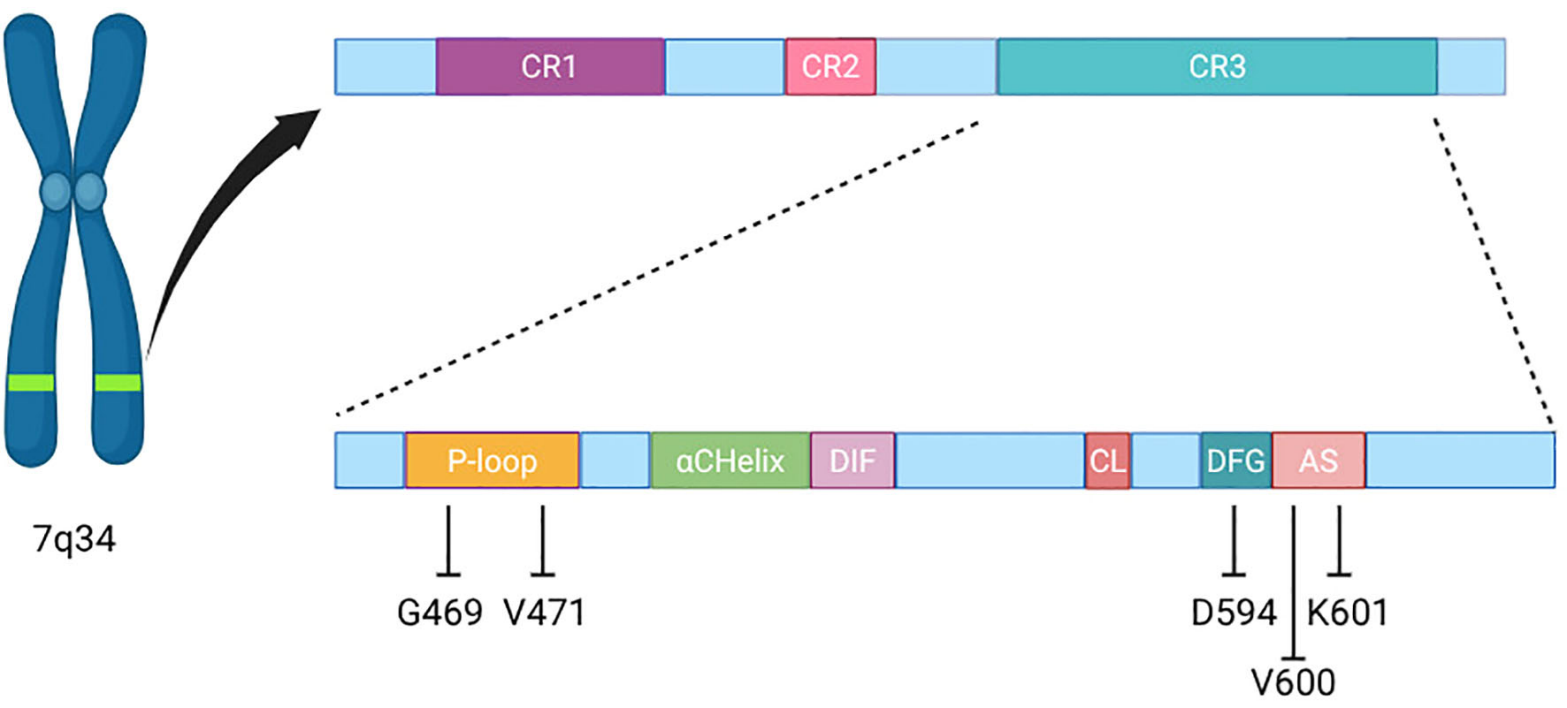
Schematic representation of BRAF gene and protein composition. CR1 contains the RAS binding domain, while CR3 is the kinase domain, which contains the P-loop, αChelix, DIF, CL, DFG motif and AS. Our patients’ mutations localisation has been highlighted, along with V600 hotspot for reference. CR, conserved regions; DIF, dimerisation interface; CL, catalytic loop; AS, activation segment.

Mutations in the v-Raf murine sarcoma viral oncogene homolog B (*BRAF*) are detected in approximately 2% of patients with advanced non-small-cell lung cancer (NSCLC) (1). Three classes of *BRAF* mutations have been identified on the basis of their biological and signaling mechanisms (2). Unlike the most common V600 mutations (class I), which produce high kinase activity and signal as monomers (3), class II mutations cause intermediate-to-high kinase activity through constitutively active dimers (4), independent of rat sarcoma virus (RAS) regulation. Furthermore, class II mutations have been subclassified based on the specific domains of the *BRAF* gene they affect: class IIa mutations occur within the activation segment (e.g., L597, T599, and K601), whereas class IIb mutations occur in the glycine-rich p-loop (e.g., G464 and G469) (**Figure 1**). On the other hand, class III mutations are located in the P-loop (G466), catalytic loop (N581) and DFG motif (D594) and are associated with a lack of or impaired BRAF kinase activity (**Figure 1**). Thus, the signaling activity with these mutations depends on activation/inactivation of other pathway protagonists such as CRAF, RAS, RTK or NF1 (4, 8).

While BRAF V600E (Class I) is the most prevalent variant, comprehensive genomic analyses now reveal that non-V600 mutations could be more frequent both considering all cancers and NSCLC, with Class II-III alterations together accounting for approximately 35% of all BRAF mutations in cancer (5).

Compared to class I, class II-III mutations are most likely observed in current or former smokers (6) and are associated with an elevated risk of developing brain metastases (p ≤ 0.01) (7); as per molecular profile, an increased incidence of co-mutations, a higher tumour mutational burden, and a lower PD−L1 expression are reported (8, 9). Unlike class I tumors, which rarely harbor concurrent MAPK pathway alterations, class II-III BRAF-mutant NSCLCs frequently co-occur with alterations in KRAS, NRAS, NF1, and CRAF (10, 11).

These clinical and pathological characteristics may, though partially, explain the significantly poorer prognosis of these patients compared to those with class I mutations (12), adding to the absence of effective targeted therapies.

In fact, while current guidelines recommend targeted therapies (dabrafenib plus trametinib or encorafenib plus binimetinib) as preferred first-line or subsequent treatment for *BRAF* V600-mutant patients (13, 14), there are no FDA- or EMA-approved targeted therapies for BRAF class II-III mutant NSCLC. Conventional type I BRAF inhibitors (e.g., vemurafenib, dabrafenib) are ineffective against class II dimers, and recent prospective trials of next-generation RAF inhibitors have yielded disappointing results so far (15). Chemotherapy and immunotherapy—despite modest and variable efficacy—remain the only reimbursed options (16, 17).

In this case series, we describe the clinical features and outcomes of a small group of patients with BRAF class II-III mutations who received off-label dabrafenib and trametinib at the IRCCS CRO Aviano, Italy, and contextualize these findings within the rapidly evolving therapeutic landscape for this underserved NSCLC subgroup.

Patients were selected from our Molecular Tumour Board historical records and clinical registry, consecutively from 2020. Costs of the off-target therapy with dabrafenib and trametinib were entirely covered by our hospital.

## Case series

### Case 1

An elderly woman, non-smoker, presented to her primary care physician towards the end of 2020 with a persistent cough unresponsive to antibiotic therapy. Total body imaging revealed a nodule in the upper lobe of the right lung along with multiple ipsilateral ground-glass opacities. An 18F-FDG PET-CT scan then showed multiple thoracic lymphadenopathies. The diagnostic bronchoscopy only allowed a cytologic diagnosis of lung adenocarcinoma with a PD-L1 Tumour Proportion Score (TPS) <1%, since the sample was not sufficient to perform next-generation sequencing (NGS).

In February 2021 a liquid biopsy was performed, not detecting *EGFR, BRAF* or *MET* alterations at the time (Institutional NGS panel). Therefore, first line treatment with carboplatin, pemetrexed and pembrolizumab was started, since the patient was symptomatic but had a performance status (PS) ECOG score 1 and only medication-controlled hypertension as comorbidity. After 4 cycles a partial response was achieved and maintenance therapy with pemetrexed and pembrolizumab was continued. Due to the sustained response and the thoracic confinement of the disease, in October 2021, after 4 cycles of maintenance therapy, radical radiotherapic treatment to the remaining lung nodule and lymphadenopathy was performed (60Gy/25fr).

After completing the treatment, pembrolizumab and pemetrexed were resumed and maintained until February 2024, when a CT scan showed progressive thoracic disease. Another liquid biopsy performed at that time showed no alterations in the *MET, EGFR* and *BRAF* genes. In order to obtain a complete molecular profile, a new bronchoscopy was then executed. The new lung tissue biopsy analyses revealed a *BRAF* K601E mutation with Variant Allele Frequency (VAF) 22%. After multidisciplinary review in the Institutional Molecular Tumour Board (MTB), an off-label authorisation for second line treatment with dabrafenib and trametinib was accorded. Therefore, in May 2024 the patient started dabrafenib and trametinib at full standard dose. The treatment is still ongoing; the patient has had a clinical improvement and CT scans reveal stable disease up until April 2025.

### Case 2

An elderly male with a history of multiple relapsing papillary urothelial carcinomas, treated with transurethral resection of the bladder (TURB) until 2023, and a previous diagnosis of prostate cancer treated with radiotherapy in 2010, underwent routine follow-up with thoracic and abdominal CT in January 2024. Imaging revealed a newly detected pulmonary lesion in the right hilar region measuring 40 × 26 mm, with initial involvement of the right lower bronchus. Additional findings included a smaller ipsilateral sub-centimetric nodule and a right hilar lymphadenopathy measuring 26 × 15 mm.

The patient was an active smoker with a cumulative exposure of 70 pack-years and had a history of arterial hypertension. In February 2024, a diagnostic bronchoscopy was performed. Histopathological examination confirmed lung adenocarcinoma (TTF-1 positive, GATA3 negative) with a PD-L1 TPS of 3–5%.

A PET-CT scan was conducted to better define the TNM stage, revealing further pulmonary and lymph node involvement, consistent with stage IV disease.

In the meantime, NGS on tissue sample identified a *BRAF* V471F mutation (VAF 34%). The case was reviewed by the Institutional MTB. Off-label first line treatment with dabrafenib-trametinib was advised in this instance due to factors such as age and PS (ECOG 1, Karnofsky 70%), which made target therapy preferable to the -standard of care- chemo-immunotherapy.

Treatment started in May 2024 at a reduced dose (dabrafenib 150 + 75 mg and trametinib 1.5 mg die) as a precaution in consideration of age. The first radiologic re-evaluation with PET-CT scan showed a decreased 18F-FDG uptake of all lesions and a dimensional reduction of some of the lymphadenopathies. Unfortunately, in November 2024 a new concomitant localised urothelial carcinoma was diagnosed. Due to rapid clinical deterioration, further diagnostic and therapeutic interventions were not feasible. The patient passed away in December 2024.

### Case 3

Another elderly male presented to the emergency department in December 2023 with severe back pain. His medical history included atrial fibrillation requiring pacemaker implantation, arterial hypertension, and hypertrophic cardiomyopathy. He was a former smoker (20 pack-years, ceased over 30 years earlier).

CT imaging revealed widespread neoplastic disease involving the liver, bones, and central nervous system (CNS), with a dominant pulmonary lesion in the left lower lobe (35 × 26 mm) and multiple bilateral pulmonary nodules, suggestive of primary lung cancer. Diagnostic bronchoscopy confirmed NSCLC and upon NGS a *BRAF* K601E mutation was found (VAF 2%).

Considering the presence of CNS involvement and actionable mutation, first-line treatment with dabrafenib and trametinib was initiated at standard dosing in December 2023, following discussion in the Institutional MTB. Denosumab and palliative radiotherapy to the hip were also commenced due to extensive bone involvement and pain.

**Figure 2:**
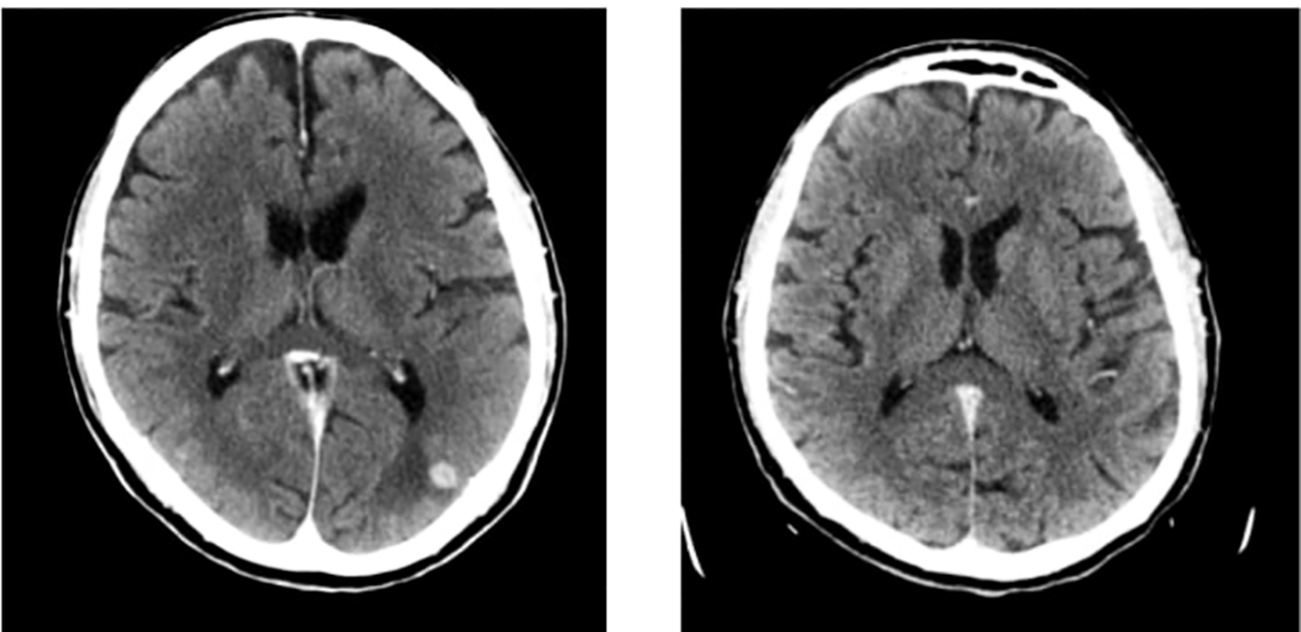
Intracranial response to target therapy: on the left basal imaging, on the right the first re evaluation.

**Figure 3:**
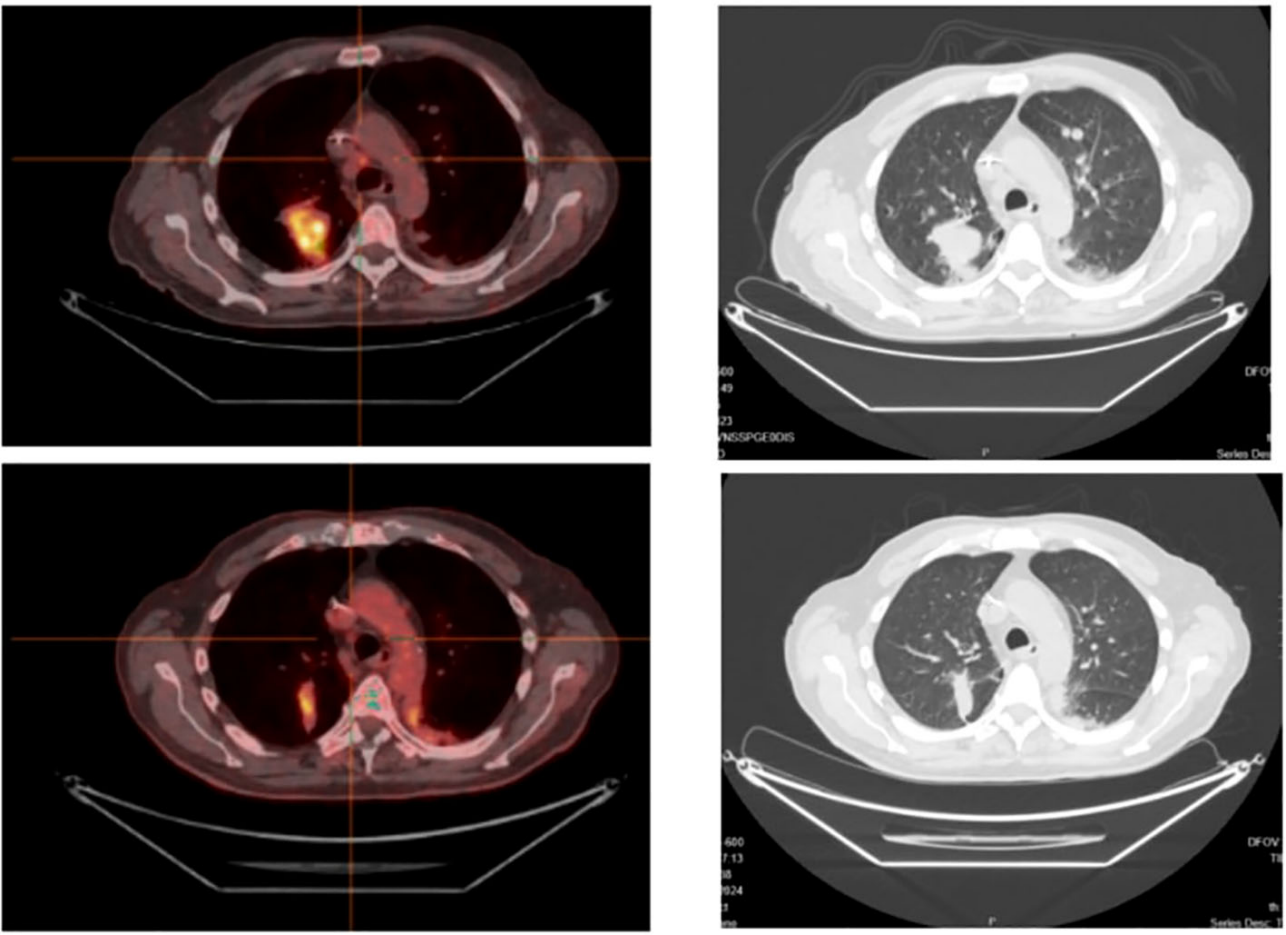
Thoracic response to targeted therapy, on a PET-CT scan (left) and CT-scan (right).

The patient showed a rapid clinical response to these treatments, and the first radiologic re-evaluation in March 2024 showed a partial response in the bones, liver and CNS lesions (**Figures 2**, **3**).

However, by May 2024, disease progression was observed, particularly in pulmonary and skeletal sites, with re-exacerbation of pain, while CNS lesions remained stable.

Palliative radiotherapy was delivered to the scapula and spine, and immunotherapy with cemiplimab was initiated based on PD-L1 expression (TPS 60%).

In August 2024, the patient presented with paraesthesia and hyposthenia in the right upper limb, and an urgent head CT scan revealed new multiple CNS metastases. However, a PET-CT scan showed partial response on all extrathoracic metastatic sites, prompting whole brain radiotherapy (WBRT) (20Gy/5fr) in September 2024, with symptomatic improvement. Cemiplimab was continued until December 2024, when further CNS progression occurred, leading to clinical deterioration. Best supportive care was provided until the patient’s death in March 2025.

### Other cases

An elderly man with no history of smoking was diagnosed with NSCLC in April 2024. NGS analysis revealed a *BRAF* K601E mutation (VAF 5.6%) and a PD-L1 TPS negative. PET-CT scan revealed stage IV disease for lymphnodal involvement. After discussion of the case in the Institutional MTB, in consideration of age and comorbidity (precedent nephrectomy with chronic kidney disease), an off label authorisation for dabrafenib and trametinib treatment was suggested. Unfortunately, the patient experienced rapid decline in general conditions after the first weeks of treatment and died before the first re-evaluation.

An elderly woman with a previous DLBCL was diagnosed with NSCLC during follow-up for the hematologic malignancy. Bilateral lung nodules constituted a stage IV disease, and NGS revealed a *BRAF* G469S mutation (VAF 19.2%). In consideration of previous treatments for the hematological disease, age and PS, an off-label request for dabrafenib and trametinib treatment was issued. The patient started treatment in July 2024 but stopped it after just a few days for grade 3 nausea and grade 1 vomiting. Stereotactic Body Radiation Therapy (SBRT) on the growing lesions was therefore performed, and the patient went on follow-up until May 2025 when progressive disease showed on the CT-scan.

A patient in his 60s was admitted to our Hospital for uncontrolled pain and important weight loss (-25 kg in 4 months) in August 2024. He was a heavy smoker and a drinker, and though diagnostic procedures were carried out revealing a NSCLC with *BRAF* D594N and *STK11* I177F mutations (VAF 5.5% and 6.5% respectively), treatment could not be started due to general conditions. He had a high burden of disease (metastasis to the bone, pleura, lymphnodes and adrenal glands) and died a few months later.

A woman about the same age presented to the emergency department in December 2024 with CNS symptoms (speech disorder and right sided weakness). She was an active smoker. Diagnostic imaging identified a single CNS lesion suspicious for secondary metastasis. Surgical resection of this lesion was performed and upon histologic examination lung adenocarcinoma with *BRAF* G469V was diagnosed (VAF 64%). Neurologic symptoms improved after surgery and radiotherapy on the surgical field. In consideration of the CNS involvement and the *BRAF* mutation, first-line treatment with dabrafenib and trametinib was recommended after MTB discussion. However, the patient refused systemic treatment.

An active smoker and young patient (<60 years old at diagnosis) received first line treatment with carboplatin and pemetrexed in a different Institution from January 2022 to September 2024 for metastatic NSCLC (for CNS and pleural involvement). Since CNS involvement at diagnosis required WBRT associated with high doses of steroids, the patient was not a candidate for first-line immunotherapy. When referred to our Centre, an NGS was performed revealing a *BRAF* G469V mutation (VAF 25%). The patient suspended pemetrexed due to renal toxicity with maintained partial response and is now continuing her follow-up scans with no signs of progressive disease so far. Therefore, systemic targeted therapy was not considered for this patient, but the case was nevertheless discussed in the Institutional MTB and dabrafenib and trametinib will be considered in case of progressive disease.

The youngest patient (<60 years old) was a male former smoker who developed haemoptysis in the summer of 2024. Upon investigation, in December a diagnosis of lung adenocarcinoma with metastasis in the brain, lymphnodes and bone was made. A *BRAF* G469A mutation was found in NGS (VAF 57.2%); however, the patient suffered a fatal stroke before any treatment could be started.

## Discussion

This is a relatively large case series of NSCLC patients with *BRAF* class II-III mutations treated -or considered for treatment- with targeted therapy in a single center.

All of the patients included had stage IV disease at diagnosis, accounting for the aggressiveness of *BRAF* mutated tumours. Principal clinical and outcome data are reported in **Table 1**.

**Table 1:** Clinicopathological characteristics and outcomes of our case series.

Number of case report	Sex	Smoking habit	PD-L1 (TPS)	*BRAF* mutations	VAF (%)	BRAFi treatment line n°	PS ECOG	PFS (months)	OS (months)	CNS involvement at diagnosis	CNS RT
1	F	never	<1%	K601E	22	2	1	14.2	53	No	No
2	M	current	3-5%	V471F	34	1	1	7.1	10.1	No	No
3	M	former	60%	K601E	2	1	1	6.1	15.1	Yes	Yes
4	M	never	<1%	K601E	5.6	1	2	1.7	3.8	No	No
5	F	former	negative	G469S	19.2	1	1	8.3	11.3	No	No
6	M	current	20%	D594N	5.5	–	3	–	7.3	No	No
7	F	current	negative	G469V	64	–	0	–	3.8	Yes	Yes
8	F	current	5%	G469V	25	–	0	–	41.6	Yes	Yes
9	M	former	20%	G469A	57.2	–	0	–	3.8	yes	No

PFS was calculated only for patients who received dabrafenib and trametinib, while OS was for the whole cohort. PS ECOG refers to start of targeted therapies if received, or baseline if not.

F, female; M, male; TPS, Tumour Proportional Score; VAF, Variant Allele Frequency; PFS, Progression Free Survival; OS, Overall Survival; CNS, Central Nervous System; RT, Radiotherapy.

The most important evidence from our small cohort was that only 3 of 9 patients were able to receive the treatment for more than 1 month. In fact, median duration of treatment was 5 cycles, while registrational trials of dabrafenib-trametinib in *BRAF* V600E mutated NSCLC report a median duration of 10–11 months (18, 19).

Best response in those who received at least one dose of treatment was stable disease, but with partial response for CNS lesions. Median progression-free survival (PFS) and overall survival (OS) in this subgroup of patients were 7.5 and 18.7 months, respectively, while in the whole series median OS was 16.6 months. These data are consistent with what is currently reported in literature for class II mutations (12, 17). The patient with class III mutation had a very short overall survival (7.3 months), again consistent with what is reported in literature, though he did not receive any systemic treatment and thus no firm conclusion can be drawn (12). We considered PFS as time from treatment start to treatment discontinuation (either for radiological or clinical progressive disease or patients decision, whichever came last) and OS as time from initial diagnosis to death or last follow-up.

A possible explanation of the worse prognosis of our patients, of which many were not even able to receive systemic treatment, is the biological aggressiveness reported with class II-III *BRAF* mutations (7). Moreover, the advanced age of most of the patients in our cohort must be taken into account, since the median age was 76 years, more advanced than the one reported in the registrational trials (18, 19). This differentiates *BRAF*-mutated cohorts from other known NSCLC actionable oncogenic alterations (AGAs) like ALK, which are more often found in younger patients (20).

Independently of the prior treatment received (including immunotherapy), patients who took dabrafenib-trametinib for more than 1 month did not experience grade 3 toxicity. In our series, ⅘ (80%) patients had a dose interruption, only 2 a dose reduction (one of them started treatment at a lower dose as a precaution due to older age, the other had grade 3 nausea in the first week and discontinued the treatment). This underlines the safety of the anti-BRAF/anti-MEK combination, not different from the registrational trial where only patients with *BRAF* V600E mutations were included (18). No additional toxicities were observed even in the patient who received immunotherapy after progression to targeted therapy.

Interestingly, only 2 patients had received another systemic treatment before dabrafenib and trametinib (one chemo-immunotherapy) with good response. Similarly, one patient was treated with immunotherapy alone after failure of targeted therapy. This still leaves open the question of which is the best sequence of treatments for these patients and the role of immunotherapy, especially in low PD-L1 or patients with CNS metastasis where its efficacy is lower (12, 21). In fact, other international experiences previously reported inconsistent benefit from immunotherapy independently of treatment sequence as well as lack of efficacy of dabrafenib and trametinib across not only different mutation classes, but also different mutation in the same class, hinting at the need for further characterisation and a better understanding of the surrounding molecular landscape with the many co-mutations (10).

As highlighted in our series, patients with CNS metastases at diagnosis may benefit from first-line targeted therapy, given its enhanced ability to penetrate the blood–brain barrier and its greater efficacy in controlling intracranial disease. In fact, intracranial efficacy of dabrafenib and trametinib in *BRAF* V600E mutated NSCLC was reported up to 80% (as ORR in patients with brain metastasis at baseline) (19), while for immunotherapy intracranial efficacy reported was 39% with chemo-immunotherapy, but no data in *BRAF* mutated patients have been reported (22) as these patients are frequently excluded from clinical trials.

Of note, 3 of the 4 patients with brain metastases at diagnosis received radiotherapy (WBRT or SBRT). One of them received the radiotherapy after dabrafenib and trametinib, and two of them never received the targeted treatment. These two patients were on corticosteroids — a contraindication to immunotherapy — at the time of first-line treatment start. This could constitute one of the criteria aiding the choice in multidisciplinary boards.

Consistent with previous reports, nearly half of our cohort had a history of either active or past smoking. PD-L1 expression was generally low, with only 1 patient having a TPS > 50%.

The G469 (class IIb) mutation was the predominant mutation (4/9), accounting for approximately half of the cases as reported in Western cohorts; the K601 (class IIa) mutation was detected in 3 patients. In previous studies (6), the sensitivity of G469-mutant cells to dabrafenib plus trametinib was inconsistent, whereas the K601 mutation was shown to predict responsiveness to BRAF inhibitors in melanoma cells. These findings suggest that class IIa-mutant NSCLC patients, in particular, could represent a promising target population for BRAF/MEK-targeted therapy.

Interestingly, one patient had a *STK11* co-mutation which is a recognized poor prognostic factor (23). However, this patient was not able to receive any treatment and no conclusion can be made on its significance when combined with a *BRAF* class II mutation. Co-mutations relations with BRAFi efficacy or prognosis in these patients are largely unexplored, though reported- especially TP53 (24, 25). Presence of more MAPK pathway co-alterations that could account for reduced sensitivity to BRAF inhibitors (10). With the advent of more extensive NGS use and broader panels, hopefully more data will be available in the future. It will be advisable to evaluate the role of these co-mutations in a prospective study, as recently done with other AGAs and targeted therapies (26).

In one of our patients, NGS performed on plasma samples failed to identify the *BRAF* driver mutation. This may depend on several factors, including the burden of the disease and the associated circulating tumour DNA shedding disease (our patient only had thoracic disease), the sensitivity and the timing of the different tests. Despite the increasing adoption of liquid biopsy in clinical practice, its concordance with tissue analysis and therefore its reliability remains subject to debate. In patients with NSCLC, the overall concordance rate between tissue and plasma NGS has been reported to be approximately 70% (27). However, specific data on *BRAF* mutations, particularly non-V600E variants, are limited and largely lacking. This paucity of evidence does not allow definitive recommendations regarding the use of liquid biopsy for this subgroup of patients.

To optimise the interpretation of NGS findings—whether derived from tissue or plasma—discussion of rare or ambiguous results within a multidisciplinary MTB is essential. It allowed us to gather all the available biological information before any clinical decision.

Unfortunately, during the time of diagnosing and treating our patients, no clinical trials in this setting were available in our centre. However, it is worth noting that most of our patients, for clinical conditions or CNS involvement, would probably not have met usual inclusion criteria. This underlines a critical unmet clinical need for more real-world-like trials, especially in these rare subgroups in which standard numerous clinical trials would probably fail due to slow accrual. Interest is growing and some promising are being published on pna-RAF inhibitors such as exarafenib (28).

Taking everything into consideration, targeted therapy cannot be consistently recommended over standard of treatment for patients with class II-III *BRAF* mutated NSCLC, in absence of prospective data. However, our case series shows that a personalised approach considering targeted therapy in the continuum of care of these patients should be taken into consideration and discussed on a case by case basis in multidisciplinary boards. Enrollment in clinical trials should be the first choice whenever possible, and efforts should be made to promote such trials in a subgroup that is proving not so rare with the advance of molecular analysis and more targeted therapies available.

## Conclusions

The combination of dabrafenib and trametinib in *BRAF* non-V600E mutated NSCLC is currently not approved in Europe. Given the limited data, the use of dabrafenib and trametinib in patients with non V600E *BRAF* mutations could be considered on a case-by-case basis, ideally within the context of a clinical trial. Here we report a relatively large BRAF class II-III mutated NSCLC case series, but more solid research is needed to better understand the best treatment course in this patient population.

Consultation within a multidisciplinary oncology team and MTB and consideration of clinical trial opportunities are recommended to explore potential treatment options.

## Data Availability

The data analyzed in this study is subject to the following licenses/restrictions: Data could only be used for these research according to patients’ informed consent. Requests to access these datasets should be directed to sara.torresan@cro.it.

## Glossary

BRAF: v-Raf murine sarcoma viral oncogene homolog B
NSCLC: Non-Small-Cell Lung Cancer
RAS: Rat Sarcoma Virus
EGFR: Epidermal Growth Factor Receptor
MET: Mesenchymal-Epithelial Transition factor
PD-L1: Programmed Death-Ligand 1
TPS: Tumour Proportion Score
NGS: Next-Generation Sequencing
ECOG: Eastern Cooperative Oncology Group (Performance Status)
PS: Performance Status
TURB: Transurethral Resection of the Bladder
PET-CT: Positron Emission Tomography-Computed Tomography
MTB: Molecular Tumour Board
VAF: Variant Allele Frequency
CT: Computed Tomography
SBRT: Stereotactic Body Radiation Therapy
WBRT: Whole Brain Radiation Therapy
CNS: Central Nervous System
PFS: Progression-Free Survival
OS: Overall Survival
BRAFi: BRAF Inhibitors
MEKi: MEK Inhibitors
STK11: Serine/Threonine Kinase 11
TP53: Tumor Protein P53
